# Health-related quality of life (HRQoL) from HIV patients’ perspective: comparison of patient-reported outcome (PRO) measures among people living with hiv (PLWH) and other chronic clinical conditions

**DOI:** 10.1186/s41687-022-00423-4

**Published:** 2022-03-26

**Authors:** C. Seguiti, P. F. Salvo, E. Di Stasio, S. Lamonica, A. L. Fedele, S. Manfrida, N. Ciccarelli, B. Corvari, C. De Luca, L. Tartaglione, D. Pitocco, R. Cauda, A. Cingolani

**Affiliations:** 1 Malattie Infettive, Università Cattolica del Sacro Cuore, Rome, Italy; 2 UOC Malattie Infettive, Infectious Disease Department, Fondazione Policlinico Universitario Agostino Gemelli IRCCS, Largo Francesco Vito 1, 00168 Rome, Italy; 3 UOC Chimica, Biochimica e Biologia Molecolare Clinica, Fondazione Policlinico Universitario A. Gemelli IRCCS, Rome, Italy; 4 Dipartimento di Scienze Biotecnologiche di Base, Cliniche Intensivologiche e Perioperatorie, Università Cattolica del Sacro Cuore, Rome, Italy; 5 Divisione Reumatologia, Fondazione Policlinico A. Gemelli IRCCS, Rome, Italy; 6 Dipartimento di Diagnostica per Immagini, Radioterapia Oncologica ed Ematologia, Fondazione Policlinico A. Gemelli IRCCS, Rome, Italy; 7 Dipartimento di Psicologia, Università Cattolica, Milan, Italy; 8 UOS Diabetologia, Fondazione Policlinico A. Gemelli IRCCS, Rome, Italy

**Keywords:** HRQoL, PLWH, PRO, HIV, Chronic illness

## Abstract

**Background:**

People living with HIV (PLWH) are generally known to suffer from a lower quality of life compared to the one of general population, but still very few is known about the self-perception of quality of life when comparing HIV to non-communicable diseases. We performed a comprehensive assessment of patient’s reported outcomes measures (PROMs) among PLWH and patients affected by other chronic conditions (OC) such as diabetes mellitus type 1, rheumatoid arthritis, breast cancer in hormonal therapy, in order to investigate differences in PROMs outcomes between PLWH and other pathologies.

**Methods:**

A cross-sectional observational study was performed by using questionnaires investigating health-related quality of life (Medical Outcomes Study Short Form 36-item Health Survey), work productivity (WPI), and global health status (EQ-5D-3L). They were administered to patients affected by chronic diseases consecutively observed at a single University Hospital during a 10 months period, with comparable disease related aspects. Logistic regression analysis was used to analyze the association between disease group (HIV vs OC) and PROMs.

**Results:**

230 patients were enrolled (89 PLWH, 143 OC). Mean age: 49 years (SD 10), mean time of disease 12 years (10), 96% were Caucasian, 35% assumed polypharmacy, 42% of male were PLWH versus 16% OC (*p* < 0.001), 19% PLWH versus 6% OC had clinical complications (*p* < 0.001). HIV infection was independently associated to a better health-related quality of life in several domains compared with the other conditions, except in mental health, whereas a worst health-related quality of life in most domains was reported by older patients and those experiencing polypharmacy.

**Conclusions:**

In this cohort of patients with chronic conditions followed within the same health setting, PLWH showed better self-reported health outcomes compared to other chronic conditions with comparable characteristics of chronicity. The potential detrimental role of older age and polypharmacy in most outcomes suggests the need of longitudinal assessment of PROMs in clinical practice.

## Introduction: Background

The continuous implementation of combination antiretroviral therapy in clinical practice, since its introduction, has completely changed the profile of PLWH, transforming HIV infection to a chronic disease and raising patients’ life expectancy up to the general population one [1]. As in other lifelong standing clinical conditions, PLWH experience multidimensional symptoms and concerns that can be burdensome on several aspects of their lives [2, 3].

The globally accepted definition of health inherited directly from the WHO Constitution perceives that only an integrated approach can grant the protection of physical, mental and social well-being [4]. Thus, a person-centered care strategy is needed as an irrevocable part of a mutual relationship whose goal is the retention-in-care of the patient, as self‐reported physical and psychological problems are associated with poorer antiretroviral therapy (ART) adherence, viral rebound, and poorer self‐rating of health [5–7]; evaluations of new treatments and interventions to improve healthcare require the measurement of HRQoL as well as clinical and viro-immunological endpoints (CD4 count, viral load, progression to AIDS) [8].

One potential solution is the ‘patient-centered approach’ incorporating person‐centered care alongside valid patient‐reported outcome measures (PROMs) [9, 10], using questionnaires as an instrument to individualize medical decision, promoting at the same time for the patient a growing self-consciousness of its main role in determining its own well-being.

While it is generally known that, even in the era of tailored medicine, PLWH have a lower quality of life when compared to the general population [11, 12], still less clear is the comparison between the perception of HIV burden and the one related to non-communicable diseases.

In this article we describe the results from our observational cohort study conducted in our University Hospital, with the goal of comparing self-perceived health status in PLWH and in patients affected from Diabetes Mellitus type one (DM1), Rheumatoid Arthritis (RA) and Breast Cancer (BC), evaluating differences in mental health, physical health, and treatment satisfaction between PLWH and other chronic conditions.

## Methods

### Study population

A cross-sectional observational study was conducted in our University Hospital Fondazione Policlinico Agostino Gemelli, Rome, Italy. The cohort of patients have been directly enrolled from those coming for follow-up visits to the Outpatients Department of this Institution from March 2019 to January 2020. Patients have been selected among those affected by the following chronic conditions: diabetes mellitus type 1, Rheumatoid Arthritis, Breast Cancer and HIV infection. Patients have been enrolled if they had the following inclusion criteria: age < 70 years, history of chronic disease > 1 years, being under stable therapy for the sub mentioned chronic disease > 6 months, life expectancy > 2 years based on judgment of the clinician, absence of main functional impairment defined by IADL score > 5. Exclusion criteria were defined by age < 18 years, inability to read and understand Italian language, inability to fill in a questionnaire, acute or unstable phase of the chronic disease, and diagnosis of dementia.

Demographics and epidemiological data have also been reported for each patient, together with information about their chronic condition, in particular about the time from diagnosis, the number of pills taken per day, the presence of complications. The presence of complications was defined according to the different diseases: any condition included in Centers for Diseases Control definition Group C for HIV infection [13]; adverse effects (AEs) associated with tamoxifen and aromatase inhibitors (AIs) in (ER)-positive Breast Cancer population, which are directly related to estrogen deprivation, with commonest effects being gynecological, cardiovascular and related to bone health [14, 15]; consequences of untreated and/or uncontrolled disease activity (such as joint deformities, cervical spine disease, rheumatoid nodules, vasculitis), extra-articular manifestations (such as respiratory and cardiac complications, eye problems) and complications due to immunosuppressive treatments (such as infections) for Rheumatoid Arthritis [16]; the following microvascular and macrovascular complications [17] for Diabetes Mellitus type I: (1) retinopathy, defined as any diabetes-related eye disease, atherosclerotic eye disease, blindness or severely impaired vision, or use of retinal photocoagulation or vitrectomy to treat diabetic retinopathy [18]; (2) neuropathy, defined according to both current guidelines and utilization of Michigan Neuropathy Screening Instrument (MNSI) physical examination score of > 2 or a previous lower limb amputation [19] and (3) nephropathy, defined as the presence of microalbuminuria (concentration of 3–30 mg/mmol or 30–300 mg/g or 30–300 mg per 24 h, or its detection at reagent strip urinalysis) and reduction Glomerular filtration rate (eGFR), estimated with the MDRD formula [20]; (4) Coronary Artery Disease (CAD), atherosclerotic cerebrovascular disease (including previous vascular ischemic stroke, transient ischemic attack [TIA] or thrombolysis/thrombectomy); (5) atherosclerotic peripheral vascular disease (femoral artery stenosis > 50% or any vascular surgery or amputation) and carotid arterial disease (defined as carotid artery stenosis > 50% or any previous carotid endarterectomy/stenting) [21].

### Patient’s reported outcomes measures

The following questionnaires exploring different aspects of living have been administered to all patients enrolled during a total observation period of ten months (March 2019–January 2020): Medical Outcomes Study Short Form 36-item Health Survey (MOS SF-36) [22, 23] for health-related quality of life, WPAI-GH [24, 25] for work productivity, EQ-5D-3L [26] for global health status.

Medical Outcomes Study Short Form 36-item Health Survey questionnaire (MOS SF-36) consists of 36 questions determining the constitution of 8 scales: physical functioning (10 questions), role limitation due to physical health (4 questions), role limitation due to emotional problems (3 questions), bodily pain (2 questions), vitality (4 questions), social functioning (2 questions), mental health (5 questions), general health (5 questions). In particular, the SF-36 version used in this study was the IQOLA SF-36 Italian Version 1.6.

The score is 0 to 100 for each scale, and higher scores indicate a better quality of life.

Work productivity and activity impairment-general health questionnaire (WPAI-GH) is aimed to explore the general burden of the disease and the impact of specific symptoms on work productivity and everyday life. Defining first the current employment situation of the subject, it proceeds through 5 more specific questions: (1) Worktime missed due to health-related reasons during the previous 7 days; (2) Worktime missed due to other reasons during the previous 7 days; (3) Effective worktime during the previous 7 days; (4) Effective burden of health-related issues on work productivity during the previous 7 days; (5) Effective burden of health-related issues on everyday life during the previous 7 days.

EuroQol questionnaire (EQ-5D-3L) explores 5 different dimensions: mobility, self-care, usual activities, pain, and anxiety/depression. For each one, the patient is asked to quantify the burden of the disease. The last question consists of a visual analog scale (VAS) that measures the perceived health-status of the patient [27].

The Health Assessment Questionnaire Disability Index (HAQ-DI) was originally developed for the assessment of functional disability in patients with RA. There are 8 sections: dressing, arising, eating, walking, hygiene, reach, grip and activities. Each section is made up of 3 or 4 questions. Scoring within each section is between 0 (no difficulties) and 3 (unable to do).

### Statistical analysis

Statistical analysis has been performed using the IBM SPSS Statistics version 20.0. All data were first analyzed for normality of distribution using the Kolmogorov–Smirnov test of normality; the related correlation coefficients and *p* values are reported in Tables 1 and 2. Continuous variables (mean age, duration of disease, mean scores for each questionnaire) were expressed as mean ± standard deviation (SD), categorical variables (gender, main diagnosis, polypharmacy) displayed as frequencies and the appropriate parametric (ANOVA with multiple comparisons Bonferroni’s correction to locate differences), or non-parametric test (Kruskal Wallis ANOVA with Dunn’s test to locate differences and χ^2^ test), was used to assess significance of the differences between subgroups. Association between variables was estimated by Spearman’s correlation coefficient. Multiple linear regressions with backward-stepwise method were also performed in order to study the relationship between PROMs and clinical and sociodemographic parameters: covariates introduced in the model were variables significantly correlated at the bivariate analysis.

**Table 1:** Socio-demographics aspects

Variable	HIV (n = 89)	DM1 (n = 30)	BC (n = 25)	AR (n = 88)	K-S test	*p*
Mean age, y (SD)	49 (10)	46 (12)^a^	54 (8)	52 (11)^a^	D = 0.99 *p* = 0.282	0.011*
Disease duration, y (SD)	14 (9)^a^	22 (14)^a,b^	2 (1)^a,b^	7 (5)^a,b^	D = 2.37 *p* < 0.001	< 0.001*
Ethnicity, %						
Caucasian	95	100	96	98.8		
Afro American	2	0	0	0		
Other	3	0	4	1.2		
Complications of the disease, n (%)	36 (40)	13 (43)	12 (48)	0 (0)		< 0.001°
Polypharmacy, n (%)	32 (36)	19 (63)	10 (40)	27 (31)		0.007°

Statistical test: *ANOVA, #Kruskal Wallis ANOVA, °X2-square or Fisher test

n Apex letters locate differences among subgroups

**Table 2:** Mean PROMs scores

PROMs	HIV	DM1	BC	AR	K–S test	*p*
SF-36						
Physical functioning (PF)	91 (17)^a^	82 (28)	79 (21)	76 (24)^a^	D = 3.84 *p* < 0.001	< 0.001^#^
Role physical (RP)	88 (29)^a^	70 (39)	59 (43)^a^	56 (42)	D = 5.21 *p* < 0.001	< 0.001^#^
Role emotional (RE)	81 (34)	69 (40)	72 (42)	64 (45)	D = 5.98 *p* < 0.001	0.085^#^
Vitality (VT)	67 (18)^a^	59 (21)	60 (20)	58 (22)^a^	D = 1.07 *p* = 0.202	0.034^#^
Mental health (MH)	69 (20)	67 (21)	72 (16)	69 (19)	D = 1.15 *p* = 0.144	0.910^#^
Social functioning (SF)	83 (20)^a^	66 (28)^a^	82 (19)	73 (23)	D = 3.05 *p* < 0.001	0.002^#^
Bodily pain (BP)	86 (22)^a^	73 (27)	73(26)	67 (24)^a^	D = 3.27 *p* < 0.001	< 0.001^#^
General health (GH)	69 (22)^a^	43 (23)^b^	65 (20)^b^	53 (22)^a^	D = 1.35 *p* = 0.051	< 0.001^#^
EQ-5D						
Score	86 (18)^a,b^	73 (20)^b^	80 (19)	72 (26)^a^	D = 2.45 *p* < 0.001	< 0.001^#^
Perceived health status, mean (SD)	79 (16)^a^	68 (13)^a^	74 (14)	76 (17)	D = 2.40 *p* < 0.001	< 0.001^#^
HAQ-DI						
Disability index, mean (0–20)	0.7 (2.7)^a^	1.1 (3.0)^b^	1.0 (2.2)^c^	4.9 (7.4)^a,b,c^	D = 5.29 *p* < 0.001	< 0.001^#^
WPAI-GH						
Impact of the disease in the workplace, mean (0–10)	0.3 (1.2)^a^	1.5 (2.0)	1.3 (2.6)	2.2 (2.8)^a^	D = 3.70 *p* < 0.001	< 0.001^#^

Statistical test: *ANOVA; ^#^Kruskal Wallis ANOVA; °X2-square

n Apex letters locate differences among subgroups

A *P* value of less than 0.05 was considered statistically significant.

## Results

A total of 230 patients have been enrolled, of whom 63% (147) were women. Among the studied population, PLWH were 89 (38.7%) of whom 27% (24) were women and 73% (65) were men; In the subgroup with DM1, 73% (22) were women and 27% (8) were men; the subgroup affected by RA was composed by 86% (76) women and 14% (12) men; 100% (25) of patients with BC were women. Socio-demographic and health-related characteristics of the enrolled patients are listed in Table 1.

### Differences in PROs questionnaires score among different diseases

PROs have been analyzed administering each questionnaire at the time of enrollment.

Table 2 shows the different performance score in questionnaires according with different diseases. At SF-36, PLWH reported significantly higher score than people affected by other diseases in all domains except for mental health, where the difference among all diseases showed no significant differences. For what concerns the other questionnaires analyzed, similar scores were obtained for perceived health status (EQ-5D-3L) and for mean disability index (HAQ-GH). Regarding the impact of disease in the workplace, as assessed by WPAI-GH questionnaire, PLWH reported a less degree of negative impact compared to the other diseases (Table 2).

### Factors associated with different quality of life

The association between specific domains of quality of life and sociodemographic and clinical characteristics of the entire studied population was shown in Table 3. At multiple regression analysis, after adjusting for the type of disease (HIV infection vs all other diseases as a whole), age, gender, ethnicity, the time from disease diagnosis, the presence of complicated disease and polypharmacy (Table 4), HIV infection resulted significantly associated with a better quality of life in terms of all domains analyzed (physical health, bodily pain, general health, physical role functioning, social role functioning and emotional role functioning domains according with SF-36, as well as the health status according with EQ-5D-3L), except for SF-36-mental health for which no disease-specific variable was associated with different quality of life. Older age was found to be associated with a worst quality of life in terms of SF36-physical health, SF36-Bodily pain, SF36- Physical role functioning and SF-36 emotional role functioning; the only other variable associated with an impaired quality of life was polypharmacy, which significantly reduced mental health, general health, vitality (according to SF36) and health status (according to EQ-5D) (Table 4).

**Table 3:** Spearman correlation coefficients

Variable	HIV	Gender	Age	Duration of the disease	Complicances	Polypharmacy
Physical health (PH)						
Correlation coefficient	− 0.40	0.23	− 0.31	0.08	0.09	− 0.10
*p*	< 0.001	< 0.001	< 0.001	0.222	0.185	0.128
Mental health (MH)						
Correlation coefficient	0.00	− 0.01	− 0.02	− 0.10	− 0.01	− 0.12
*p*	0.979	0.910	0.725	0.148	0.938	0.062
Bodily pain (BP)						
Correlation coefficient	− 0.38	0.23	− 0.28	0.01	0.08	− 0.13
*p*	< 0.001	< 0.001	< 0.001	0.900	0.226	0.041
General health (GH)						
Correlation coefficient	− 0.29	0.15	− 0.12	− 0.08	0.07	− 0.15
*p*	< 0.001	0.021	0.066	0.224	0.291	0.022
Physical functioning (PF)						
Correlation coefficient	− 0.37	0.23	− 0.22	0.08	0.13	− 0.07
*p*	< 0.001	< 0.001	0.001	0.242	0.045	0.292
Vitality (VT)						
Correlation coefficient	− 0.18	0.14	− 0.12	0.00	0.06	− 0.15
*p*	0.005	0.029	0.064	0.949	0.333	0.023
Social functioning (SF)						
Correlation coefficient	− 0.18	0.12	− 0.04	− 0.07	0.05	− 0.05
*p*	0.007	0.068	0.565	0.267	0.470	0.452
Emotional functioning (EF)						
Correlation coefficient	− 0.16	0.12	− 0.14	− 0.04	0.04	− 0.12
*p*	0.014	0.080	0.039	0.564	0.587	0.060

**Table 4:** Significative (*p*< 0.05) correlations between PROMs and socio-demographic and clinical characteristics at multiple regression analysis

PROMs	Variable	b	SE	*p*
SF-36				
Physical health (PH)	Age*	− 0.48	0.13	< 0.001
	HIV**	− 12.32	2.89	< 0.001
Mental health (MH)	Polypharmacy^#^	− 5.05	2.60	0.054
Bodily pain (BP)	HIV**	− 16.12	3.16	< 0.001
	Age*	− 0.54	0.14	< 0.001
General health (GH)	Polypharmacy^#^	− 5.16	3.09	0.01
	HIV**	− 18.18	3.10	< 0.001
Physical functioning (PF)	Age*	− 0.73	0.22	0.007
	HIV**	− 27.6	4.98	0.001
Vitality (VT)	Polypharmacy^#^	− 6.36	2.73	0.021
	HIV**	− 8.97	2.75	0.001
Social functioning (SF)	HIV**	− 11.08	3.11	< 0.001
Emotional functioning (EF)	HIV**	− 14.35	5.39	0.008
	Age*	− 0.55	0.24	0.026
EQ-5D				
Score	HIV**	− 6.03	1.22	< 0.001
	Age*	0.33	0.15	0.025
	Duration of the disease	− 0.32	0.16	0.045
	Polypharmacy^#^	− 11.3	3.2	< 0.001
Perceived health status	Polypharmacy^#^	− 5.15	2.09	0.014
	HIV**	− 5.52	2.11	0.014

other variables in the model: gender, ethnicity, time from chronic condition diagnosis, presence of complications related to chronic condition

^*^For each year older

**vs other condition

^#^Any drug other than drugs for primary chronic condition

## Discussion

The results of this study documents that in a cohort of patients with different chronic pathologies with comparable characteristics of chronicity, followed in the same hospital care setting, albeit by different doctors, over the same period, PLWH have a better self-reported state of health than people living with other chronic pathologies such as diabetes mellitus type I, rheumatoid arthritis, breast carcinoma in hormone therapy. This difference is reported for most of the quality-of-life domains analyzed.

Each domain examined in our study showed how PLWH outcome results in being the most satisfying compared to all other chronic diseases considered, while people living with RA resulted as the worst outcome group primarily in domains related to physical functioning, physical pain, and limitation related to emotional factors.

The only domain in which PLWH did not have the best outcome is the mental health in which, however, PLWH reported scores perfectly comparable with patients affected by other diseases. Our results in such sphere are concordant with the findings of Miners et al., that highlighted how PLWH have frequent mental health disturbances, due to depression and anxiety [11]. A peculiar role in mental health of PLWH is potentially attributable to the social stigma: in the work of Nobre et al., the social dimension is crucial in determining the experience of a worse perception of illness related to HIV infection, and the multidimensional phenomenon of self-stigma [28], which is a direct consequence of the stigmatization of the illness in the society, can lead to a discrediting self-perception and guilt that easily flow into psychiatric disorders. The introduction of routinely QoL questionnaires and PRO assessment in clinical practice for PLWH and its continuous monitoring, results have proved to be extremely useful not only in providing a prompt detection and intervention on such mental disturbances, but also in serving as a sort of snapshot of the social stigma and its perception. The trajectories designed by such studies involving PRO could help in defining areas of intervention for future global health programs aimed to break the social barriers.

According to our results, another factor related to an impaired quality of life is polypharmacy, which also has an impact on mental health. Efforts in minimizing the burden of pills in PLWH must be concordant with the increment of life-expectancy in such patients, where HIV infection overlaps with other clinical conditions requiring daily medications. When possible, treatment simplification strategies in viro-immunologically stable individuals can have a key role in improving health perception in PLWH, although the impact of the drugs used for the comorbidities that may afflict the HIV patient may have a greater impact on the drug burden than the burden of the antiretroviral therapy itself [29, 30].

In the years 1990–2000, several scientific works compared patient outcomes in terms of health-related quality of life in HIV infection and other chronic conditions [30–33]. However, although they represent fundamental works for the application and dissemination of PROMS in clinical practice, especially with regard to the management of PLWH, they refer to a very different historical period from the current one in which the focus on PLWH in patients regularly taking antiretroviral therapy has shifted mostly from issues related to acuity (clinical progression, survival, side effects of drugs…) to issues related to long-term chronic management (comorbidity, long-term adherence, degree of satisfaction with treatment, disease burden, etc.). Hays et al. [33] reported how patients with AIDS had worse physical functioning and partially emotional functioning, than those with other chronic diseases such as epilepsy, gastroesophageal reflux disease, clinically localized prostate cancer, clinical depression, diabetes.

To our knowledge, the comparison in terms of self-reported health outcomes between patients with HIV infection and other chronic diseases has not been frequently explored in the era of combined antiretroviral therapy. The work of Engelhard et al. [12] has been the first study in literature aimed to analyze health-related QoL in PLWH comparing it to other chronic conditions in the ART era, relying on the previously dominant thought that health-related QoL of PLWH is lower than the one in general population. The study’s results revealed how PLWH experience a worsening of QoL related to mental health directly connected with the patient’s immunological status, while people with AIDS experience a worse QoL related to health condition connected to prolonged life therapy and the presence of comorbidity. Although representing a fundamental study, the main limitation of Engelhard et al. lies in the collection of the information regarding people with chronic conditions, which have been obtained by previous studies [34–37] and not by direct enrollment from the same center, thus including different patients in different time frames and geographical locations, and distinct healthcare contexts. By consequence, alterations encountered may not only be reflection of the disease itself, but it may be connected to factors regarding place, time and healthcare assistance. Differently from what reported by Engelhard et al. study, the cohort of patients in our study has been actively recruited, and it is homogeneous both in time frame and healthcare facility. Our study gathered information on patients treated between March 2019 and January 2020 in the same hospital thus minimizing the variability related to healthcare assistance, patients’ management, and logistic differences.

Although consecutively observed patients were enrolled in our study, the proportion of PLWH with complicated disease (defined as a previous diagnosis of AIDS) was significantly higher than for other diseases. Although the CDC classification of 1993 appears to be rather outdated and does not reflect the current clinical reality of HIV infection, we do not currently have any other standardized definition of a complicated patient. It is certainly not possible to standardize the definitions of complicated disease among such different diseases, but it can be excluded that the better health-related quality of life reported by PLWH compared to other diseases is due to the selection of an "easier" population to manage. What emerges from this consideration is that the determinants of health-related quality of life for PLWH today may not be related to previous history of disease, but rather to the difficult handling of current therapy and advancing age.

We can obviously highlight some limitations in our study. First of all, the necessity of using generic questionnaire, non-HIV specific, aimed at identifying comparable items for each disease due to the willingness of including clinical conditions other that HIV in our study population, could have led to an overestimation of health-related QoL in PLWH [38, 39]. Moreover, despite the strenuous attempt of homogeneity in our study population demographic characters, PLWH were found to be globally younger, thus probably affecting positively the perception of health-status comparing to other clinical conditions. We are aware that HIV infection has its own peculiarities as a chronic pathology that make it difficult to compare with the others, but the criteria for inclusion in the study have been selected with the objective of balancing the potential differences inherent in the individual pathologies.

Notwithstanding our University Hospital is a reference one for many acute and chronic pathologies in central-southern Italy, in particular for those considered in the present manuscript, it is not possible to exclude a priori a selection bias whereby more severe patients may be followed at the center. For these reasons it cannot be excluded the possibility that in other care systems, where some chronic diseases have a different severity, the results may be different.

## Conclusion

In conclusion, in a cohort of patients with chronic conditions followed within the same health setting and in the same period of time, PLWH showed better self-reported health outcomes compared to other chronic conditions with sufficiently comparable characteristics of chronicity. Although the results of this study cannot be generalized and exported to other clinical settings, they do suggest that the implementation of PROs measurement in the routine management of PLWH patients, could improve the clinical assistance of PLWH, empowering patient’s self-consciousness of their clinical status, well-being and self-efficacy [40] and could be a good tool to identify the right allocation of resources in chronic diseases.

## Data Availability

Data and material are available upon request to corresponding author.
